# Clinical Performance of Oral Anticoagulants in Elderly with Atrial Fibrillation and Low Body Weight: Insight into Italian Cohort of PREFER-AF and PREFER-AF Prolongation Registries

**DOI:** 10.3390/jcm11133751

**Published:** 2022-06-28

**Authors:** Vincenzo Russo, Emilio Attena, Matteo Baroni, Roberta Trotta, Marius Constantin Manu, Paulus Kirchhof, Raffaele De Caterina

**Affiliations:** 1 Cardiology Unit, Department of Translational Medical Sciences, University of Campania “Luigi Vanvitelli”-Monaldi Hospital, 80131 Naples, Italy; emilioattena@hotmail.it; 2 Cardiologia 3–A. De Gasperis Cardio Center, ASST GOM Niguarda Ca’Granda, Piazza dell’Ospedale Maggiore 3, 20162 Milan, Italy; bimatteo@gmail.com; 3 Medical Affairs Department, Daiichi Sankyo, 00142 Rome, Italy; roberta.trotta@daiichi-sankyo.it; 4 Daiichi Sankyo Europe GmbH, Zielstattstr. 48, 81379 Munich, Germany; mariusconstantin.manu@daiichi-sankyo.eu; 5 Institute of Cardiovascular Sciences, University of Birmingham, Birmingham B15 2TT, UK; p.kirchhov@uke.de; 6 Chair of Cardiology, University of Pisa, Lungarno Antonio Pacinotti, 43, 56126 Pisa, Italy; raffaele.decaterina@unifi.it

**Keywords:** oral anticoagulants, elderly, safety, body weight, atrial fibrillation, effectiveness

## Abstract

Background: Elderly patients are at high risk of both ischaemic and bleeding events, and the low body weight is considered a risk factor for major bleeding in atrial fibrillation (AF) patients on anticoagulation therapy. The aim of our study was to compare the safety and effectiveness of non-vitamin K antagonist oral anticoagulants (NOACs) versus well-controlled vitamin-K antagonists (VKA) therapy among AF patients aged >75 years and with a body weight <60 kg in a prospective registry setting. Methods: Data for this study were sourced from the Italian cohorts of PREFER in AF and PREFER in AF PROLONGATION registries. The occurrence of a composite of stroke, transient ischemic attack and systemic embolism (thromboembolic events) was the primary effectiveness endpoint. The occurrence of major bleeding was the primary safety endpoint. All-cause hospitalizations and all-cause death were the secondary endpoints. The net clinical benefit (NCB) was calculated in order to obtain an integrated assessment of the anti-thromboembolic and pro-haemorrhagic effects of NOACs vs. VKA. Results: Overall, 522 patients were included; 225 were on treatment with NOACs and 317 patients with VKA. The NOAC group more frequently featured a higher BMI and a higher prevalence of history of stroke/TIA and insulin-requiring diabetes; conversely, heart failure and chronic liver disease were less frequent in the NAOC group. In the unmatched study population, 18 patients (3.6% in the NOAC vs. 3.2% in the VKA group, *p* = 0.79) experienced thromboembolic events; 19 patients (1.78% in the NOAC vs. 4.73% in the VKA group, *p* = 0.06) experienced major bleeding events; and 68 patients were hospitalized during the follow-up (9.3% vs. 14.8%, *p* = 0.06). After balancing for potential confounders by using the 1:1 propensity score matching technique, 426 patients (213 on NOAC and 213 on VKA) were selected. We found no significant differences in terms of thromboembolic events (3.76% vs. 4.69%, *p* = 0.63), major bleeding events (n: 1.88% vs. 4.22%, *p* = 0.15) and hospitalizations (9.9% vs. 16.9%, *p* = 0.06) between NOAC vs. VKA matched population. Based on these incidences, we found a positive net clinical benefit (+1.6) of NOACs vs. VKAs. Conclusions: These real-world data suggest the safety and effectiveness of using NOACs in elderly patients with low body weight.

## 1. Introduction

Low body weight frequently occurs in elderly patients and is a risk factor for major bleeding in patients with atrial fibrillation (AF) on anticoagulation therapy—both with vitamin K oral anticoagulant (VKA) [1] and with the non-vitamin K antagonist oral anticoagulants (NOACs) [2]. Moreover, low body weight has been associated with a higher risk of thromboembolism compared with non-low body weight patients [3]. Patients with body weight <60 kg were clearly less represented in randomized clinical trials (RCT), occurring in <10% of the study populations [4]; however, the efficacy and safety of NOACs compared with warfarin in patients with low body weight seem to be consistent with the overall findings of the pivotal registration trials. There are still few clinical data on the safety and effectiveness of NOACs in elderly patients with AF and low body weight [5,6]. We therefore extracted data on AF patients aged ≥75 years and with body weight <60 kg from two real-world prospective registries, evaluating the clinical performance in terms of safety and effectiveness of NOACs vs. VKAs.

## 2. Methods

### 2.1. Study Population

Data from this study were pooled from two prospective, observational, European registries of anticoagulants in AF: the Prevention of thromboembolic events–European Registry in Atrial Fibrillation (PREFER in AF) [7] and the Prevention of thromboembolic events–European Registry in Atrial Fibrillation PROLONGATION (PREFER in AF PROLONGATION) [8], which enrolled AF patients between January 2012 and January 2013 and between June 2014 and May 2015, respectively, and followed for 1 year. The clinical endpoints were recorded in the registries as clinically judged by the investigators. All AF patients from Italy aged ≥75 years and with body weight <60 kg who received a treatment with VKAs or NOACs were included. Potentially eligible VKA and NOAC-treated patients were propensity score-matched to generate an analysis cohort with minimal differences in baseline characteristics. The occurrence of stroke, transient ischemic attack (TIA), systemic embolism (SE), major bleeding (MB) and hospitalizations were assessed. All-cause death was recorded. All patients provided written informed consent for the inclusion the Prefer AF registry and the present study was conducted in accordance with the Declaration of Helsinki and its amendments. Ethical approval was obtained according to the local regulations of each participating centre.

### 2.2. Definitions

MB included fatal bleeding or bleeding in a critical organ or clinically relevant bleeding with haemoglobin decrease ≥2 g/dL, consistent with the definition from the International Society on Thrombosis and Haemostasis [9]. Stroke was defined as the abrupt onset of a focal neurologic deficit lasting >24 h. TIA was defined as focal neurologic deficit lasting <24 h. Systemic embolic event was defined as abrupt arterial insufficiency with documentation of an arterial occlusion.

### 2.3. Outcomes

The occurrence of MB is the primary safety endpoint. The composite of stroke, TIA and SE is the primary effectiveness endpoint. Secondary endpoints were all-cause hospitalizations and all-cause death.

#### Statistical Analysis

Descriptive results for continuous variables were expressed as mean ± standard deviation (SD); those for categorical variables as frequency and percentage. The Student’s *t*-test and the χ^2^ test were used to compare the continuous and categorical variables, respectively. Propensity score matching (PSM), with ratio of matching was 1:1, was used to balance differences in baseline characteristics between patients receiving NOACs versus those receiving VKAs. The model included the following variables: age, sex, weight, arterial hypertension, diabetes mellitus, heart failure, coronary heart disease, peripheral artery disease and chronic kidney disease. The net clinical benefit (NBC) with NOACs vs. VKAs was calculated among matched cohorts with the following formula: NCB = (thromboembolic events incidence rate with VKAs − thromboembolic events incidence rate with NOACs) − weighting factor × (intracranial haemorrhage incidence (ICH) rate with NOACs − ICH incidence rate with VKAs), where we used a weighting factor of 1.5, as previously described [10,11]. The difference in rates of outcome events among matched groups during the follow-up period was assessed by the Kaplan–Meier method by means of the log-rank test. A two-sided *p*-value less than 0.05 was considered significant for all tests. All statistical analyses were performed using RStudio (RStudio Team (2016). RStudio: Integrated Development for R. RStudio, Inc., Boston, MA, USA, available online: http://www.rstudio.com/ accessed on 15 February 2022.

## 3. Results

A total of 522 AF patients with age >75 years and weight <60 kg on treatment with a NOAC (*n* = 225) or a VKA (*n* = 317) were recorded in the two registries. Their baseline characteristics are summarized in Table 1. No data on international normalized ratio (INR) control in VKA-treated patients were available. However, INR control was assessed by collecting the last 3 INR measurements prior to enrolment. An adequate INR control (i.e., at least 2 of 3 INR values in the therapeutic range) was demonstrated in 72% of the VKA group.

**Figure 1:**
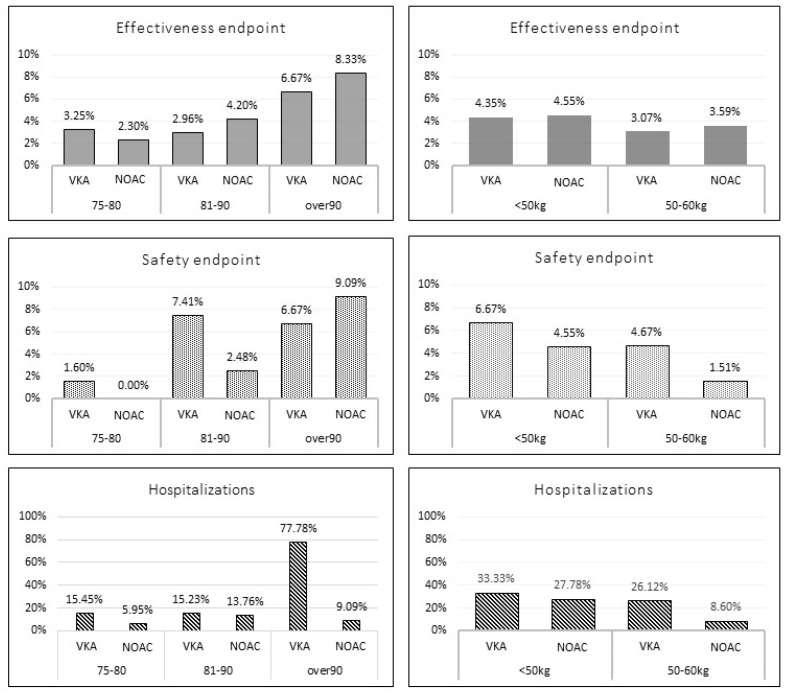
Incidence of the outcome events according to age and weight stratification among unmatched study population. VKA: vitamin K antagonist; NOAC: direct oral anticoagulant.

The NOAC group was more likely to have a higher BMI and a higher prevalence of history of stroke/TIA and insulin-dependent diabetes; conversely, heart failure and chronic hepatic disease were less frequent. No significant difference in follow-up duration between the two groups were found (369 ± 31 vs. 369 ± 12 days; *p =* 0.3). Among the overall population, 19 patients (4 in NOAC vs. 15 in VKA group) experienced MB events; a trend of reduction of MB overall incidence was reported among NOAC group compared to VKA group (1.78% vs. 4.73%; *p* = 0.06). A total of 18 patients (8 in the NOAC vs. 10 in the VKA group) experienced TE events; no significant difference in the overall incidence of TE events was shown between the two groups (3.6% vs. 3.2%, *p* = 0.79). Sixty-eight patients were hospitalized during the follow-up (21 in the NOAC vs. 47 in the VKA group); a trend of lower rate of hospitalizations was present in the NOAC group compared to the VKA group (9.3% vs. 14.8%; *p* = 0.06). A total of 7 patients (6 in the NOAC vs. 1 in the VKA group) died; no significant difference in the overall incidence of death was shown between the two groups (2.8% vs. 0.4%, *p* = 0.12). Figure 1 shows the outcome events stratified according to age (75–80 yrs.; 81–90 yrs.; and >90 yrs.) and weight (<50 kg and 50–60 kg). No statistically significant differences were found across the subgroups, except for a statistically significant lower rate of all-cause hospitalizations among patients with weight 50–60 kg (8.6% vs. 26.1%; *p* = 0.0004) and those over 90 years (9.1% vs. 77.8%; *p* = 0.004) for NOACs vs. VKAs.

### Matched Population

After balancing for potential confounders, a population of 426 patients (213 on NOAC and 213 on VKA) were selected. Among the NOAC group, 48 patients (22.5%) were taking apixaban, 59 (27.7%) dabigatran and 106 (49.8%) rivaroxaban. Baseline characteristics of the study population are summarized in Table 2.

**Figure 2:**
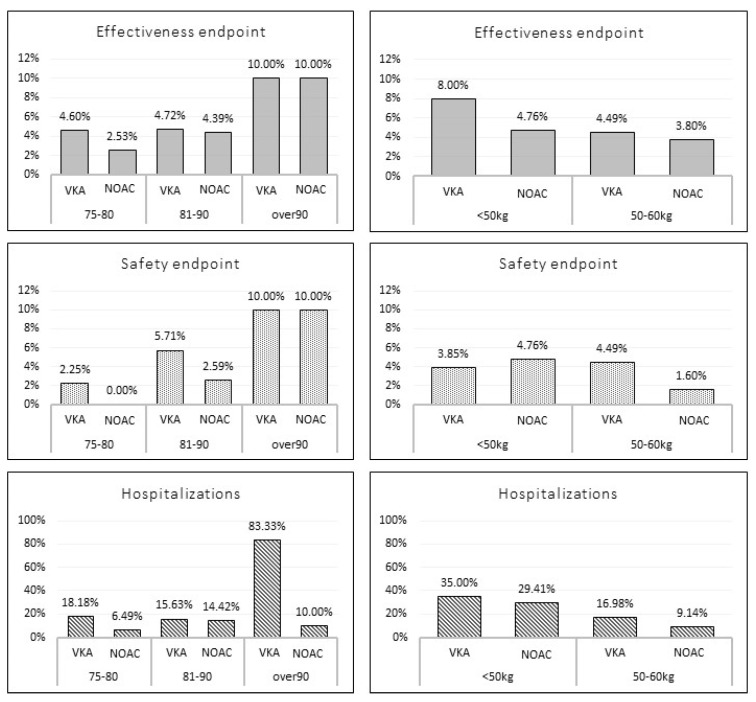
Incidence of the outcome events according to age and weight stratification among matched study population. VKA: vitamin K antagonist; NOAC; non-vitamin K oral anticoagulant.

No significant differences were found in terms of TE (3.8% vs. 4.7%, *p =* 0.63), MB events (1.9% vs. 4.2%, *p* = 0.15) and hospitalizations (9.9% vs. 15.9%; *p* = 0.06) overall incidences between NOAC vs. VKA matched populations. Figure 2 shows the incidence of outcome events stratified according to age (75–80 yrs.; 81–90 yrs.; and >90 yrs.) and weight (<50 kg and 50–60 kg). No statistically significant differences were found across the subgroups.

**Figure 3:**
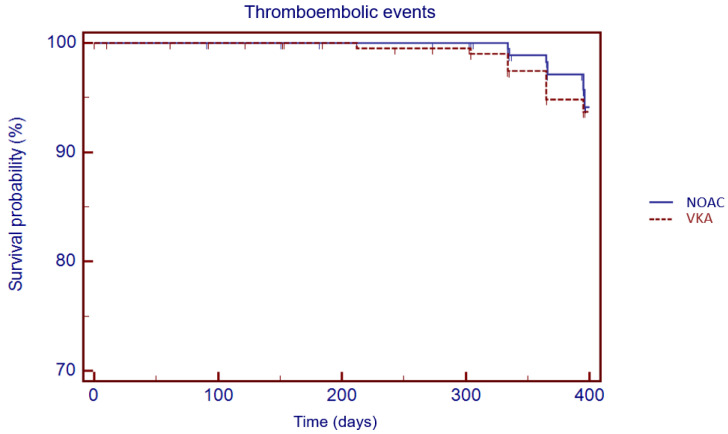
Kaplan–Meier cumulative probability of thromboembolic event-free survival in NOAC and VKA treatment group.

**Figure 4:**
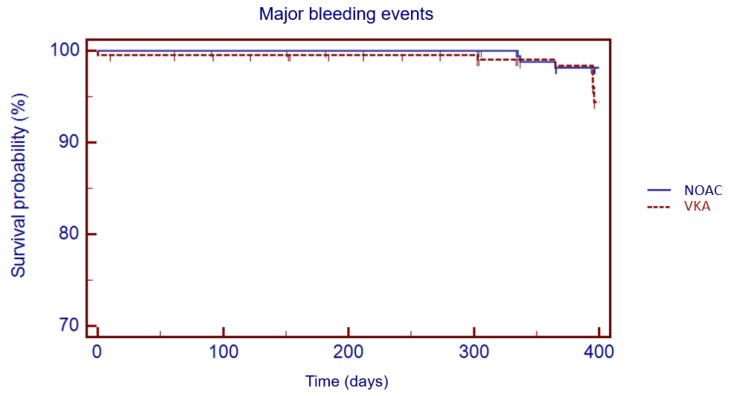
Kaplan–Meier cumulative probability of major bleedings event-free survival in NOAC and VKA treatment group.

**Figure 5:**
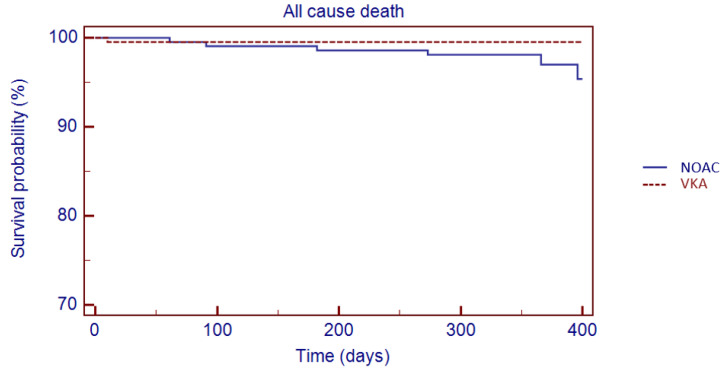
Kaplan–Meier cumulative probability of all-cause death event-free survival in NOAC and VKA treatment group.

The Kaplan–Meier analysis showed no significant difference in 1 year survival from TE events (log rank: 0.08, *p* = 0.78), major bleeding (log rank: 1.05, *p* = 0.30) and all-cause death (log rank: 3.49, *p* = 0.06) in VKA versus NOAC group (Figure 3, Figure 4 and Figure 5).

**Figure 6:**
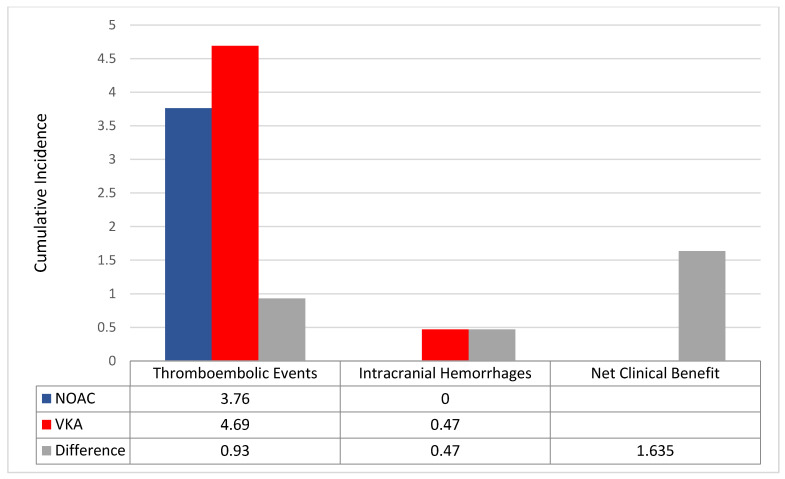
Incidence rates of thromboembolic events and intracranial haemorrhages between the two matched population. Differences (Δ) between incidence rates were used to calculate the net clinical benefit (NCB). VKA: vitamin K antagonist; NOAC: non-vitamin K oral anticoagulant.

The NOAC group featured a positive net clinical benefit vs. VKAs (+1.6) between the two matched population (Figure 6).

## 4. Discussion

The main results of the present analysis are that the overall incidence of both MB and TE events among elderly with atrial fibrillation and low body weight on oral anticoagulation therapy was high. NOACs showed a numerically lower incidence of both TE and MB events compared to VKAs across different weight and age, except for those aged >90 years. Among patients with body weight <50 kg, NOACs showed numerically higher MB events. NOACs also showed a better net clinical benefit than VKAs, mainly driven by the numerically lower rate of in TE events and intracranial haemorrhages; numerically lesser all-cause hospitalizations were shown among the NOAC vs. the VKA group.

The high overall incidence of MB events (3.64%) in our study population was expected, being both advanced age and the low body weight associated with a heightened risk for bleeding during anticoagulant treatment [1,12]. In contrast, the expected lower risk of TE events [13] seems to be attenuated by the concomitant low body weight, which has been associated with a paradoxical increase in thromboembolism risk among AF patients on oral anticoagulant treatment [3].

To date, few data on the safety or efficacy of NOACs among elderly patients with low body weight are available [4,5]. In a large Asian cohort including 21.679 AF patients with median age of 73 years and low body weight (≤60 kg) on oral anticoagulation therapy, NOACs, compared with VKAs, were associated with a lower risks of ischemic stroke, intracranial bleeding, hospitalization for major bleeding and all-cause death in patients with low body weight (≤60 kg); moreover, a consistent trend was observed in patients with extremely low body weight (<50 kg), except for hospitalization for gastrointestinal bleedings [4]. Recently, among an Italian cohort of octogenarians with atrial fibrillation and low body weight [5], Russo et al. showed a significantly lower incidence of all-cause death in patients on NOACs versus VKAs, confirming the evidence of a mortality advantage that emerged from randomized clinical trials (RCTs) on NOACs vs. VKAs in AF patients [13].

Among our study subgroup aged >90 years, no difference in thromboembolic and bleeding events was shown between NOACs and VKAs. Moreover, NOACs showed a numerically higher rate of major bleeding events among elderly AF patients with body weight <60 kg. This evidence suggests that caution should be adopted in considering NOACs “per se” a more effective and safer treatment than VKAs among very elderly patients with AF when a very low body weight and advanced age coexist [14,15,16].

Patients on NOACs showed a lower hospitalization rate compared with those on VKAs. The much lower rate of hospitalization among patient aged >90 years could not be justified by a lower incidence of MB or TE events alone, since it was similar across NOACs and VKAs matched groups. The conservative management of NOAC related complications [17] and the fear of intracranial haemorrhages in elderly patients on VKA therapy [18] might in part justify our results; however, further analyses of the Prefer in AF registry are needed to explore the reasons for hospitalizations [19].

### Limitations

No outcome data on patients receiving edoxaban were provided. A direct comparison among NOACs was not performed, due to the small number of endpoint events during the observation period. Data about the appropriateness of NOACs dosage were not provided; however, we cannot exclude that octogenarians with AF may have received an inappropriate dose of NOAC [20]. No specific information about OAC adherence and persistence were available. Finally, our results were adjusted for possible confounding variables, but residual confounders cannot be excluded (i.e., previous stroke or TIA, history of bleeding and severely reduced clearance of creatinine).

## 5. Conclusions

Our study provides evidence of the safe and effective use of the NOACs among elderly patients with low body weight. This evidence was mainly accounted for by a numerical reduction in both TE events and intracranial haemorrhages. Moreover, the hospitalization rate was lower among patients on NOACs.

## Figures and Tables

**Table 1:** Clinical characteristics of the study population.

Variables	NOAC Group *n*: 225	VKA Group *n*: 317	*p* Value
Age, years	82.46 ± 4.85	82.21 ± 4.87	0.62
Sex (males)	21 (9.3%)	49 (15.4%)	0.03
Weight, kg	55.73 ± 4.46	54.76 ± 5.07	0.02
Height, cm	157.99 ± 6.34	158.42 ± 6.46	0.53
BMI (kg/m^2^)	22.40 ± 2.35	21.90 ± 2.27	0.02
CHADS_2_	2.7 ± 1.24	2.56 ± 1.21	0.56
CHA_2_DS_2_-VASc	4.7 ± 1.34	4.68 ± 1.38	0.19
HAS-BLED	2.26 ± 0.9	2.32 ± 1.03	0.12
Arterial Hypertension, n (%)	174 (77%)	227 (72%)	0.13
Diabetes Mellitus, n (%)	34 (15%)	40 (13%)	0.40
Dyslipidaemia, n (%)	75 (33%)	90 (28%)	0.14
COPD, n (%)	19 (8.4%)	40 (13%)	0.12
Heart Failure, n (%)	55 (24%)	113 (35%)	0.05
CHD, n (%)	43 (19%)	66 (21%)	0.6
PAD, n (%)	14 (6.2%)	14 (4.4%)	1
Previous Stroke, n (%)	40 (17.7%)	38 (12%)	0.06
Previous TIA, n (%)	43 (19%)	31 (9.7%)	0.002
Previous Major Bleeding, n (%)	3 (1.3%)	7 (2.2%)	0.45
CKD, n (%)	56 (25%)	61 (19%)	0.11
Chronic Hepatic Disease	1 (0.4%)	8 (2.5%)	0.06
Ejection fraction (%)	58.93 ± 10.12	57.25 ± 12.08	0.11
Creatinine Clearance (mL/min)	36.02 ± 9.36	29.23 ± 9.99	0.0003

Mean ± SD unless otherwise stated. BMI: body mass index; COPD: chronic obstructive pulmonary disease; CHD: coronary heart disease; PAD: peripheral artery disease; TIA: transient ischemic attack; CKD: chronic kidney disease.

**Table 2:** Clinical characteristics of matched study population.

Variables	NOAC Group *n*: 213	VKA Group *n*: 213	*p* Value
Age, years	82.57 ± 4.82	82.15 ± 4.93	0.37
Sex (males)	19 (8.9%)	32 (15%)	0.06
Weight, kg	55.79 ± 4.46	55.11 ± 4.82	0.13
Height, cm	158.06 ± 6.39	158.47 ± 6.58	0.50
BMI (kg/m^2^)	22.41 ± 2.38	22.01 ± 2.26	0.07
CHADS_2_	2.66 ± 1.21	2.61 ± 1.24	0.67
CHA_2_DS_2_-VASc	4.66 ± 1.29	4.54 ± 1.52	0.38
HAS-BLED	2.29 ± 0.6	2.38 ± 1.16	0.31
Arterial hypertension, n (%)	164 (77%)	149 (70%)	0.10
Diabetes Mellitus, n (%)	31 (14.5%)	22 (10%)	0.16
Dyslipidaemia, n (%)	73 (34%)	55 (26%)	0.07
COPD, n (%)	18 (8.4%)	26 (12%)	0.22
Heart Failure, n (%)	53 (25%)	67 (31%)	0.13
CHD, n (%)	41 (19%)	47 (22%)	0.44
PAD, n (%)	14 (6.5%)	10 (4.7%)	0.42
Previous Stroke, n (%)	39 (18%)	25 (12%)	0.08
Previous TIA, n (%)	43 (20%)	22 (10%)	0.004
Previous Major Bleeding, n (%)	3 (1.4%)	3 (1.4%)	1
CKD, n (%)	53 (25%)	42 (20%)	0.22
Chronic Hepatic Disease	1 (0.5%)	6 (2.8%)	0.06
Ejection fraction (%)	59.16 ± 10.15	57.31 ± 12.13	0.11
Creatinine Clearance (mL/min)	36.27 ± 9.54	30.47 ± 10.99	0.008

Mean ± SD unless otherwise stated. BMI: body mass index; COPD: chronic obstructive pulmonary disease; CHD: coronary heart disease; PAD: peripheral artery disease; TIA: transient ischemic attack; CKD: chronic kidney disease.

## Data Availability

The data presented in this study are available on request from the corresponding author.
